# Supplementary material for: Exploratory bioinformatics investigation reveals importance of “junk” DNA in early embryo development
Source: BMC Genomics. 2017 Feb 23;18:200. doi: 10.1186/s12864-017-3566-0 (PMC5324221; doi:10.1186/s12864-017-3566-0)
Supplement: Additional file 2: — Tables S7-S10 and Figures S1-S41. It also includes a list of main observations from our open-ended analysis. Table S7. Association of repeats and transcription factor binding sites with expression change in 2-cell mouse embryo. Table S8. Repeats significantly associated with zygote genome activation in human. Table S9. Repeat elements associated with gene expression in zebrafish. Table S10. Confirmation of association of repeats with ZGA gene expression in zebrafish. These are followed by Figures S1-S41. (PDF 1849 kb) [file 12864_2017_3566_MOESM2_ESM.pdf]

Exploratory bioinformatics investigation reveals importance of “junk” DNA in early embryo development

Steven Xijin Ge

Summary of observations

- There is a small number of genes upregulated in early 2C, which are enriched with noncoding snoRNAs and microRNAs. Major reprograming of gene expression occur at mid 2C stage during zygotic genome activation (ZGA), when maternal RNAs are reduced and thousands of essential genes are upregulated.
- Among the 10 gene clusters defined based on the dynamics of gene expressing during PD, a group of 3310 maternal transcripts are rapidly reduced and maps predominately to maternal allele. Others are either transiently expressed, or activated and maintained during different stages.
- Single-cell RNA-seq data also enables the estimation of retrotransposons activity during PD. Their expression patterns mirror those of regular genes.
- The promoters of group B genes are enriched in MT2_Mm and other long terminal repeats (LTRs), while these of group D genes are overrepresented in MT2C_Mm LTRs. Short Interspersed Nuclear Elements (SINEs) such as B1 and B2 are enriched in the promoters of group C, D, F, and G genes.
- ERVL family LTRs are associated with mid 2C gene expression possibly by serving as promoters to strongly induce transient expression of many nearby protein-coding genes and retro-genes. These genes show expression pattern similar to those of LTRs. The regulatory roles of some of the LTRs has been studied previously[1, 2]. One expressed LTR codes microRNA (miR-1194) from several genomic loci.
- Oocyte-specific homeobox (Obox) transcription factors (TFs), which are poorly understood but are among the most drastically regulated TFs during PD, might induce transient gene expression during PD. Obox factors may be the upstream regulator of Zscan4, as part of the cascades of TFs. This is supported by their expression profiles and enrichment of TFBS in some LTRs upstream of genes transiently expressed during PD.
- Presence of mouse B1 retrotransposons, a Short Interspersed Nuclear Element (SINE) similar to human Alu, in promoters are strongly associated with the upregulation of thousands of genes during ZGA. B2 elements have significant but weaker correlation. The association is independent of CpG dinucleotides and CpG islands, thus less likely to be explained entirely by DNA methylation. Genes with multiple B1 and B2 elements in promoters are more highly expressed and more likely to be evolutionarily conserved; many code for structural components of basic cellular machinery. It appears that B1 and B2 elements promote expression of housekeeping genes similar to enhancers. It will be interesting to study if the absence of some selected B1 elements from the genome will affect the expression of downstream genes and embryo development.
- B1 and B2 elements are stronger predictor of gene expression in embryonic stem cells (ESCs) than in differentiated progenitor cells and adult tissues. Similar trend is observed in induced pluripotent stem (iPS) cells.

- In human, gene expression change during ZGA is also correlated with the distribution of Alu SINE elements in a dosage dependent fashion. In bovine, it is the tRNA family of SINE repeats. Thus SINEs may play a role in ZGA in mammals. In Zebrafish, an AT-rich DNA transposon is a predictor of gene expression after ZGA.
- The frequency of various transposable elements (TEs) in the mouse genome follows log-normal distribution. Some LTRs are enriched in the promoter regions in a strand-specific fashion. Many are associated with PD gene expression. Most retrotransposons located in introns are more likely to be on the opposite strand. It is likely some originated from introns spliced from RNAs. Simple repeats, DNA transposons, and LINEs are enriched in introns. Most LTRs are depleted from introns. SINEs are enriched in promoters, introns and downstream regions. While genes with SINEs in promoter are more likely to code for proteins that constitute intracellular parts, LINEs are more often found in promoters of genes related to G-protein coupled receptor activity, an extracellular signaling process.
- Systematic analysis of the promoters of co-regulated genes at various stages identified many TFs that might be involved in gene regulation during PD, including some well-known regulators of early embryo development such as Oct4, KLF4, and Sox2. NOBOX and MECOM may drive gene expression at early 2C. Several homeobox factors (SEBOX, EMX1, HOXA7, and HOXA13) are downregulated together with their target genes during 2-4C stage. At blastocyst stage, I divided the cells are into three groups and identified enrichment of (a) SOX2, OCT4, HESX1, SIX1, and CDX1 binding sites in promoters of genes upregulated in a group of cells resemble epiblast, (b) CUX1, MSX1, ISX, SOX17 binding sites in promoters of genes upregulated in cells that may give rise to primitive endoderm, and (c) MLX, MSX2, and STAT6 bindings sites in promoters of genes induced in trophoblast-like cells.
- This study also demonstrates that single-cell RNA-seq is a powerful method for study biological pathways, especially when applied to normal developmental processes
- There is limited evidence that organisms with longer generation time tend to have bigger genomes, supporting the notion that the expansion of transposable DNA elements may be a necessary mechanism for genotypic diversity and adaptation in slow-reproducing organisms. This may shed some light on the “C-value paradox” that highlights the lack of association of genome size with organismal complexity.

Supplementary Tables S7-

Table S7. Association of Repeats and transcription factor binding sites with fold-change in 2-cell stage mouse embryo.

Rep. Class	Rep. Family	Repeat/TF Name	Strand	Effect Estimate	P Value	FDR	Target genes
Simple_repeat		(TG)n	A	-0.19	9.7E-04	2.5E-02	**816**
Simple_repeat		(TG)n	S	-0.17	2.5E-03	4.7E-02	**855**
Simple_repeat		(TTCC)n	S	**0.98**	7.7E-05	2.8E-03	50
Low_complexit		GA-rich	A	-0.47	3.5E-04	1.0E-02	145
Low_complexit		T-rich	S	-0.33	5.1E-03	7.5E-02	171
SINE	Alu	B1_Mm	A	0.35	4.9E-07	**2.4E-05**	**577**
SINE	Alu	B1_Mm	S	0.50	8.8E-12	**8.6E-10**	**530**
SINE	Alu	B1_Mur1	A	0.21	1.6E-03	3.5E-02	**611**
SINE	Alu	B1_Mur2	S	0.24	2.9E-03	4.9E-02	**437**
SINE	Alu	B1_Mur4	A	0.25	1.7E-04	5.6E-03	**644**
SINE	Alu	B1_Mur4	S	0.37	2.7E-07	**1.4E-05**	**537**
SINE	Alu	B1_Mus1	A	0.37	2.9E-16	**8.5E-14**	**1369**
SINE	Alu	B1_Mus1	S	0.28	1.0E-08	**6.8E-07**	**1174**
SINE	Alu	B1_Mus2	A	0.52	1.0E-25	**6.1E-23**	**1083**
SINE	Alu	B1_Mus2	S	0.30	5.4E-08	**3.2E-06**	**953**
SINE	Alu	PB1D9	S	0.24	2.6E-03	4.8E-02	**442**
SINE	B4	RSINE1	S	-0.16	3.3E-03	5.3E-02	**899**
LINE	L1	L1MB5	A	**-0.87**	1.6E-03	3.5E-02	16
LINE	L1	L1MD	S	**-1.10**	3.8E-03	5.9E-02	15
LINE	L1	Lx10	S	**1.41**	2.8E-03	4.8E-02	14
LINE	L1	Lx2B2	S	**2.13**	1.3E-09	**9.6E-08**	22
LINE	L1	Lx4A	S	**0.98**	1.2E-03	2.8E-02	21
LTR	ERVL	MERVL_2A-int	S	**0.94**	1.5E-03	3.4E-02	15
LTR	ERVL	MT2_Mm	S	**1.73**	2.3E-04	7.3E-03	15
LTR	ERVL	MT2B	A	0.34	1.8E-03	3.7E-02	**217**
LTR	ERVL	MT2B1	S	0.48	4.1E-03	6.2E-02	63
LTR	ERVL	MT2B2	S	**0.87**	2.1E-05	**9.0E-04**	54
LTR	ERVL	MT2C_Mm	S	**2.02**	1.2E-12	**1.4E-10**	27
LTR	ERVL-	ORR1A3-int	A	**1.47**	1.1E-04	3.9E-03	11
LTR	ERVL-	ORR1B1	S	**1.01**	2.8E-05	1.1E-03	38
LTR	ERV1	RLTR14-int	S	**0.96**	2.8E-03	4.8E-02	12
LTR	ERVK	RLTR19	S	**0.86**	1.9E-03	3.9E-02	19
LTR	ERVK	RMER17C-int	A	**0.89**	7.9E-06	**3.6E-04**	12
LTR	ERVK	RMER19B	S	**1.02**	3.8E-04	1.1E-02	16
TFBS		c-Myc		0.33	5.2E-13	**7.6E-11**	**2067**
TFBS		E2f1		0.17	1.6E-11	**1.4E-09**	**7588**
TFBS		n-Myc		0.11	3.3E-03	5.3E-02	**3918**
TFBS		STAT3		0.28	7.3E-04	2.0E-02	**439**
CpG island		CpG island		-0.09	2.3E-03	4.5E-02	**9105**

Table S8. Repeats significantly associated with zygote genome activation in human.

	Rep. Family	Rep. Class	Strand	Coefficient	P value	FDR	Target Genes
AluJb_a	Alu	SINE	A	0.21	1.1E-03	7.6E-02	764
AluJb_s	Alu	SINE	S	0.24	2.0E-04	2.1E-02	797
AluSp_s	Alu	SINE	S	0.35	1.1E-03	7.6E-02	367
AluSq2_a	Alu	SINE	A	0.31	1.3E-03	7.6E-02	435
AluSx_s	Alu	SINE	S	0.39	5.4E-08	**2.9E-05**	732
AluSz6_s	Alu	SINE	S	0.57	7.1E-05	1.3E-02	205
AluY_a	Alu	SINE	A	0.30	1.2E-04	1.7E-02	630
MER5B_a	hAT-Charlie	DNA	A	-0.60	5.3E-04	4.8E-02	113

Table S9. Repeat elements associated with gene expression in zebrafish.

Rep. Name	Rep. Family	Rep. Class	Estimate	Std. err.	FDR	Target genes	A/T content
DNA-2-19_DR	DNA	DNA	0.27	0.08	6.88E-02	17	76.2%
DNA11TA1_DR	TcMar-Tc1	DNA	0.13	0.02	1.88E-09	813	70.1%
EnSpm-N4_DR	En-Spm	DNA	0.23	0.04	7.45E-06	251	73.1%
EnSpm-N7_DR	En-Spm	DNA	0.24	0.06	1.93E-02	123	61.1%
ERV1-N1-LTR_DR	ERV1	LTR	0.25	0.07	1.93E-02	71	63.3%
ERV1-N4-LTR_DR	LTR	LTR	0.47	0.13	1.93E-02	21	61.8%
Gypsy50-LTR_DR	Gypsy	LTR	0.76	0.22	3.65E-02	11	60.6%
hAT-N36_DR	hAT	DNA	0.35	0.08	6.99E-04	25	61.9%
piggyBac-N3_DR	PiggyBac	DNA	0.35	0.08	3.29E-03	59	67.6%

Table S10. Confirmation of association with repeats in zebrafish.

Rep. Name	Rep. Family	Rep. Class	Estimate	Std. err.	FDR	Target genes
**Anes et al. data [3], 3.5 hpf vs. 128 and 256 cell stage**						
CpG_island			0.18	0.03	6.55E-10	1127
DNA11TA1_DR	TcMar-Tc1	DNA	0.13	0.03	1.29E-04	417
EnSpm-N3_DR	En-Spm	DNA	0.58	0.16	2.09E-02	17
EnSpm-N4_DR	En-Spm	DNA	0.45	0.06	9.64E-10	128
EnSpm-N7_DR	En-Spm	DNA	0.34	0.09	2.09E-02	67
ERV1-N4-LTR_DR	LTR	LTR	0.60	0.18	7.37E-02	11
Mariner-N7_DR	TcMar	DNA	0.24	0.07	3.16E-02	96
**Anes et al. data [3], 5.3hpf vs. 3.5hpf**						
DNA-2-18_DR	En-Spm	DNA	-0.60	0.18	6.67E-02	126
DNA-TA-2_DR	DNA	DNA	0.55	0.14	1.73E-02	150
DNA11TA1_DR	TcMar-Tc1	DNA	0.40	0.08	5.65E-05	417
EnSpm-N4_DR	En-Spm	DNA	0.75	0.18	9.07E-03	128
TDR16	DNA	DNA	0.40	0.11	5.18E-02	236
**Harvey et al. data [4], 6hpf vs. 3.5hpf**						
DNA11TA1_DR	TcMar-Tc1	DNA	0.20	0.04	5.65E-06	813
EnSpm-N4_DR	En-Spm	DNA	0.27	0.08	5.13E-02	251
ERV1-N8-LTR_DR	ERV1	LTR	-0.90	0.26	5.30E-02	19
hAT-N47_DR	hAT	DNA	-0.34	0.09	2.99E-02	82
LSU-rRNA_Hsa	rRNA	rRNA	0.79	0.21	2.99E-02	24
TDR16	DNA	DNA	0.20	0.05	2.99E-02	441
**Harvey et al. data[4], 3.5hpf vs. 64 cell**						
DNA-2-19_DR	DNA	DNA	0.27	0.08	6.88E-02	17
DNA11TA1_DR	TcMar-Tc1	DNA	0.13	0.02	1.88E-09	813
EnSpm-N4_DR	En-Spm	DNA	0.23	0.04	7.45E-06	251
EnSpm-N7_DR	En-Spm	DNA	0.24	0.06	1.93E-02	123
ERV1-N1-LTR_DR	ERV1	LTR	0.25	0.07	1.93E-02	71
ERV1-N4-LTR_DR	LTR	LTR	0.47	0.13	1.93E-02	21
Gypsy50-LTR_DR	Gypsy	LTR	0.76	0.22	3.65E-02	11
hAT-N36_DR	hAT	DNA	0.35	0.08	6.99E-04	25
piggyBac-N3_DR	PiggyBac	DNA	0.35	0.08	3.29E-03	59

Supplementary Figures

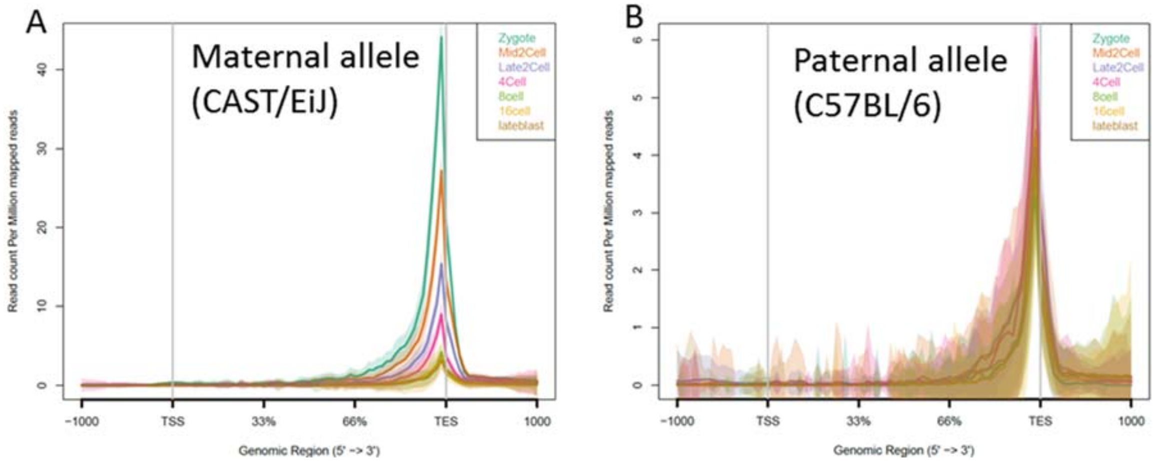

Figure 1. Density of allele-specific read mapping for cluster A genes. Note that the scales are different in (A) and (B). More reads are mapped to the maternal allele (A) than paternal allele (B). Also expression levels are reduced in the order from zygote to blastcyst as these transcripts are degraded (A). TSS, transcription starting site. TES, transcription ending site.

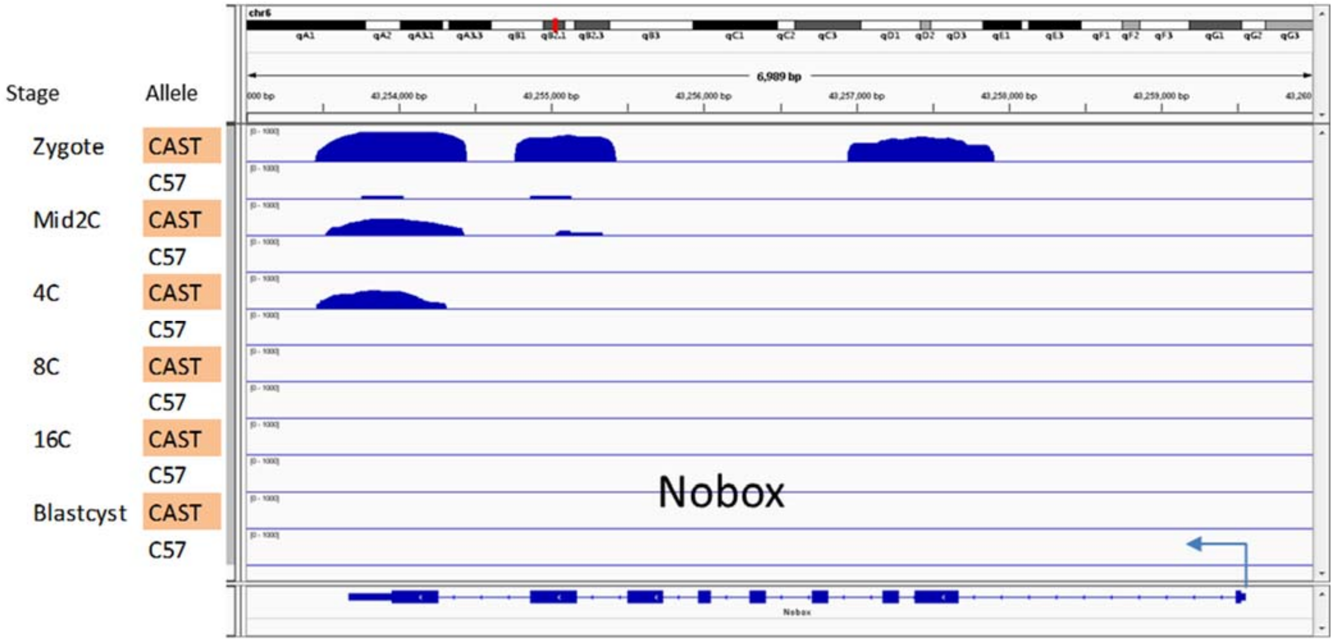

Figure 2. Allele-specific mapping of reads in the Nobox gene locus. As a member of the A cluster, these transcripts originated mostly from the maternal (CAST/Eij) allele. Also the expression is highest in zygote, and much reduced in 2C and 4C stage as the read density is shown on a log-scale. These transcripts are likely carried over from oocyte and undergo decay in early embryo development.

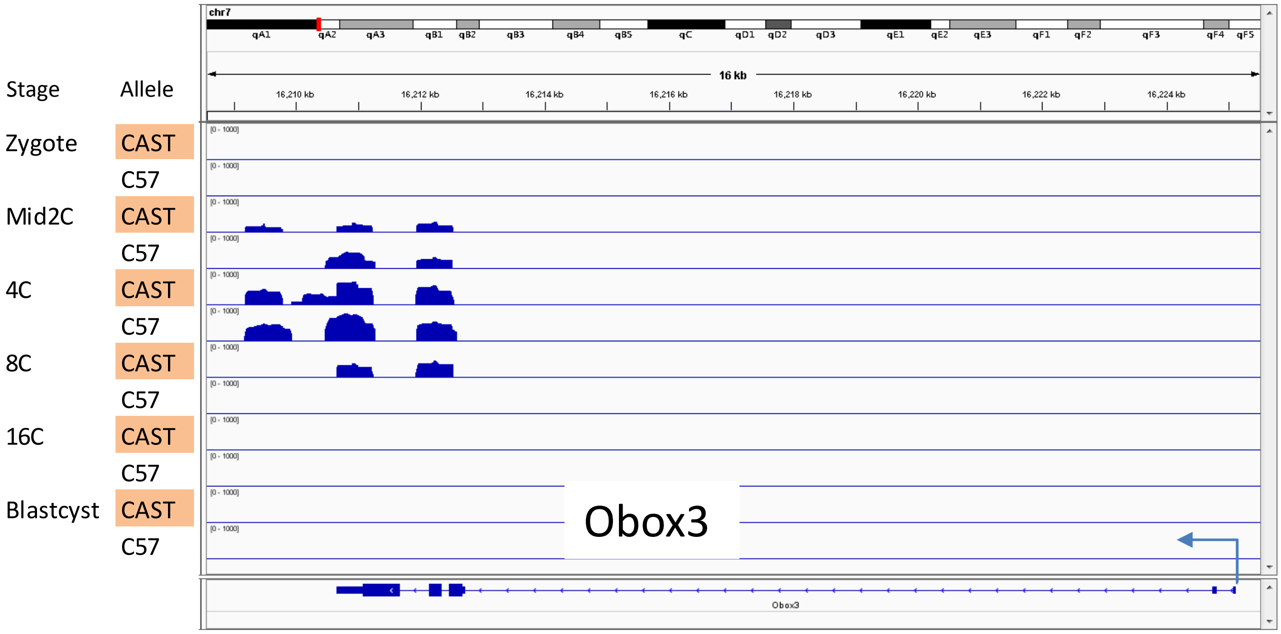

Figure 3. Allele-specific mapping in the Obox3 locus. This gene is transiently expressed during the 2C to 4C stage from both alleles.

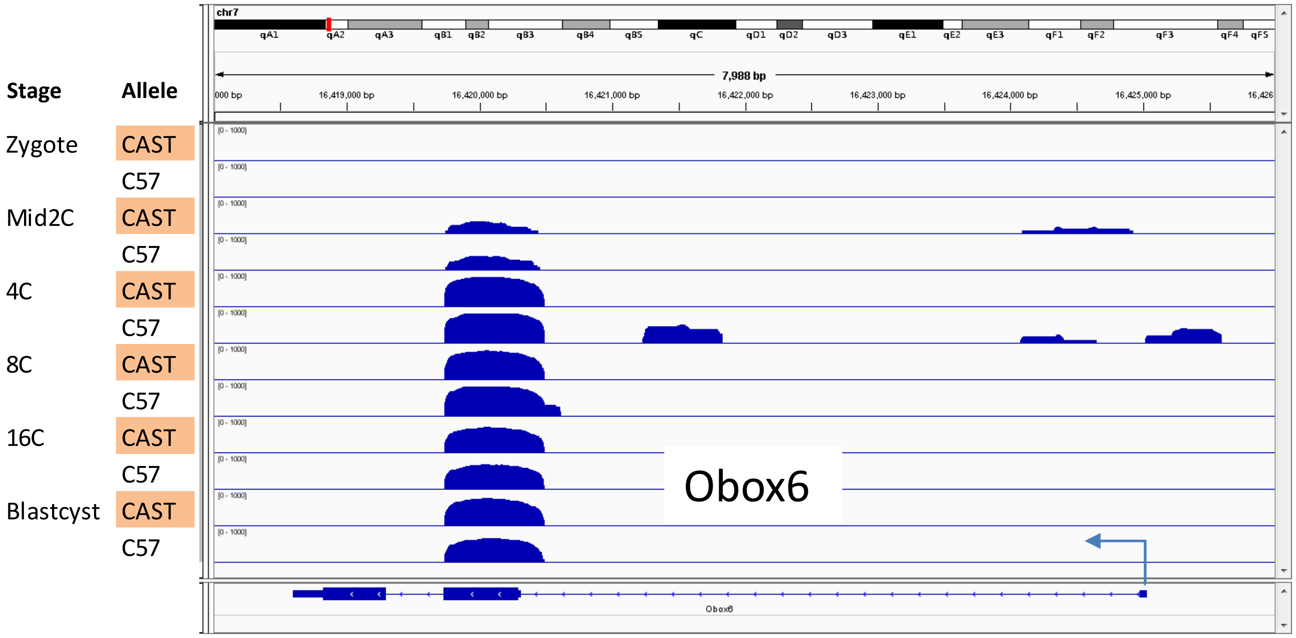

Figure 4. Allele-specific mapping in the Obox6 locus. This gene is transcribed from both alleles from the mid-2C stage to blastocyst.

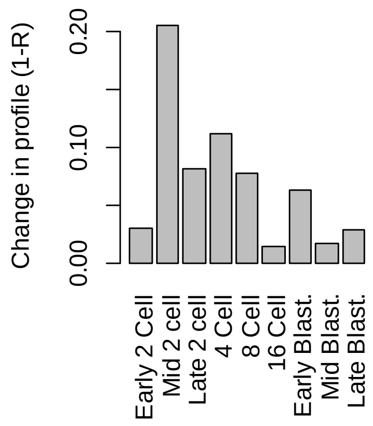

Figure 5. Change in global expression profile as defined by (1-R), where R is the Pearson's correlation coefficient. A lower bar indicates global expression profile is more similar to the previous stage.

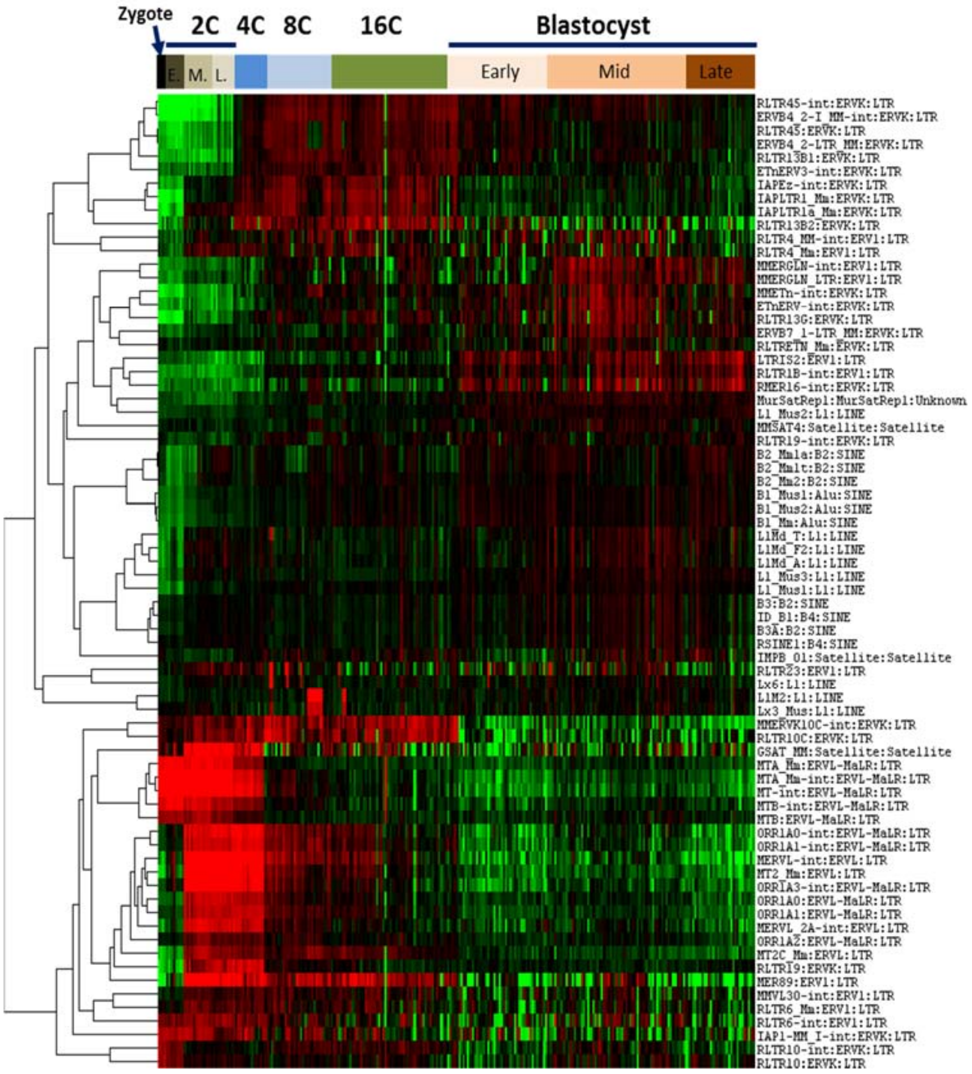

Figure 6. Expression pattern of retrotransposon during PD. Red denotes expression level is higher than average across samples. Green indicates below average gene expression.

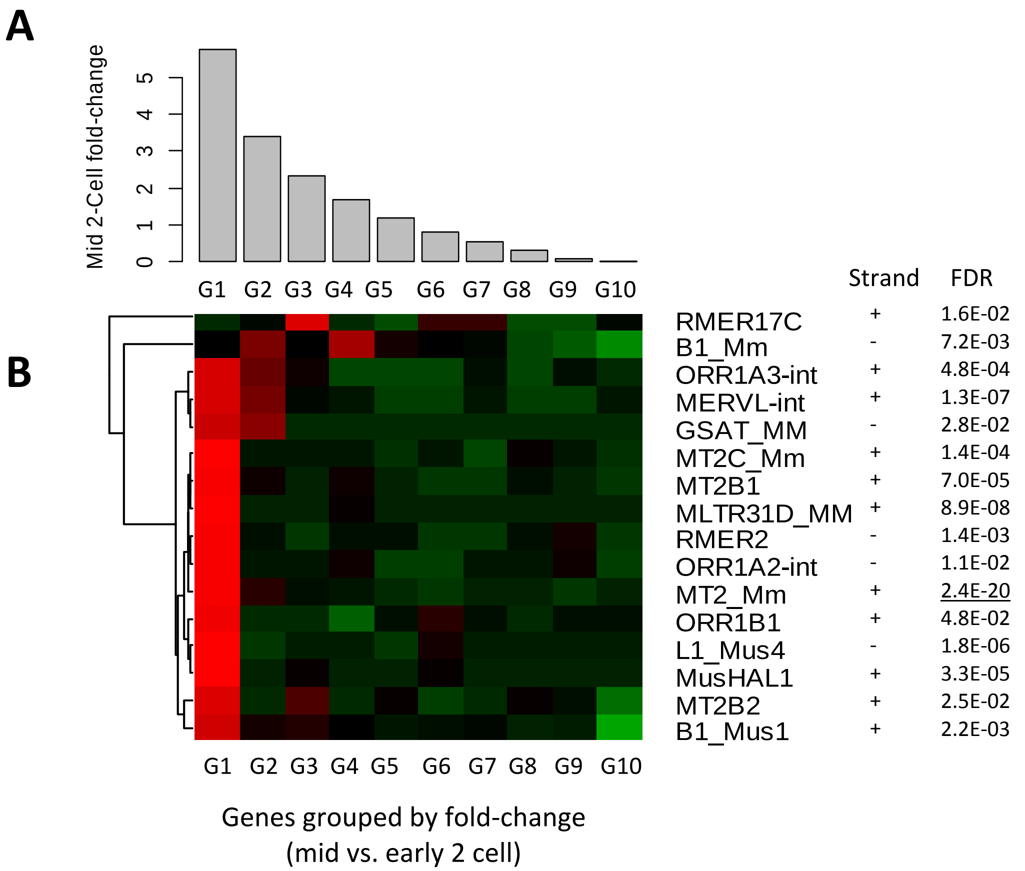

Figure 7. Enrichment of LTR elements in highly expressed genes in mid 2-cell stage. A) Genes are divided into groups based on fold-change at mid 2C. B) Enrichment of repeats in the promoters of genes are marked as red. As the first stage of zygotic genome activation, genes upregulated at mid 2-cell stage are more likely than expected to contain LTR elements in their promoters in the same DNA strand.

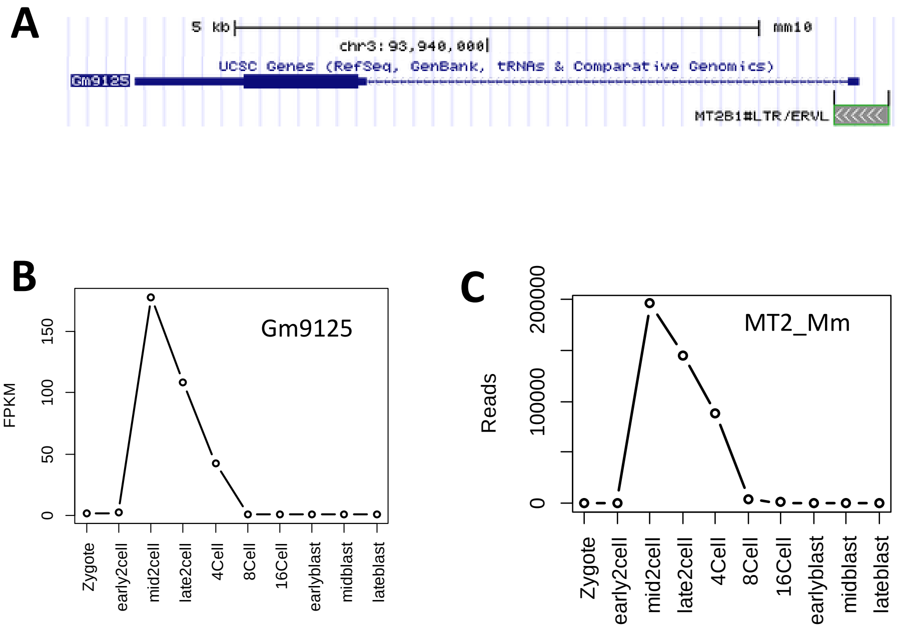

Figure 8. LTRs serve as promoters of nearby genes. A) The protein coding gene Gm9125 starts at the middle of LTR element, MT2B1. B) This gene is highly induced at mid 2-cell stage, but expression diminishes at 8 cell stage. C) MT2_Mm retrotransposons are expressed in a very similar pattern.

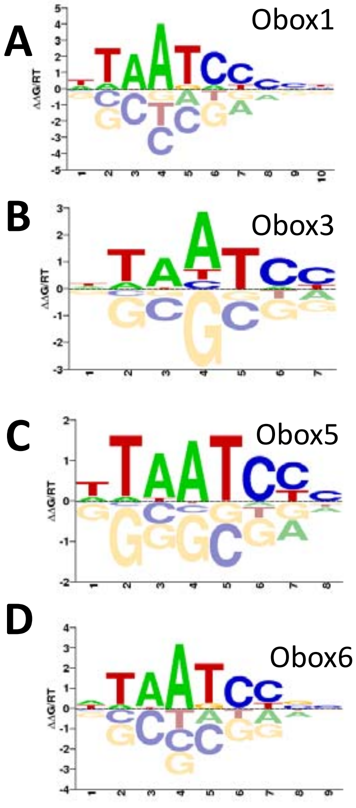

Figure 9. Obox family binding motifs according to CIS-BP.

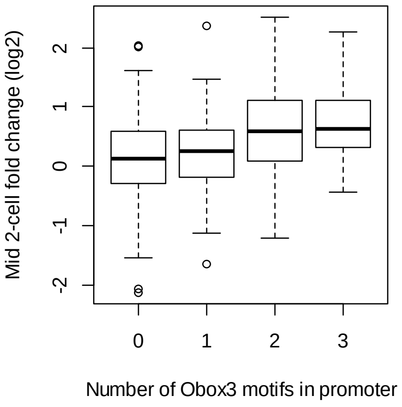

Figure 10. Genes with more Obox3 binding motifs in promoters tend to be expressed at higher levels.

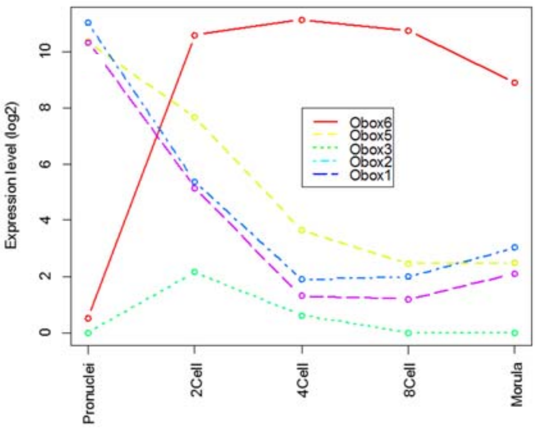

Figure 11. Expression of Obox genes in the dataset of [5].

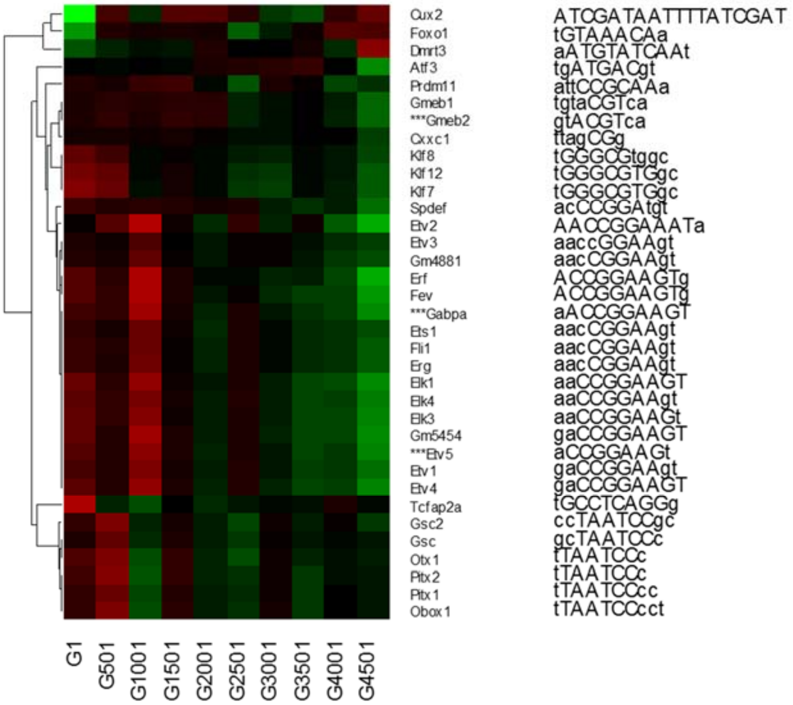

Figure 12. Identification of motifs and TFs enriched in genes upregulated in 2C using data from [5]. This agrees with the result reported in the main paper, especially in the "TAATC" motif.

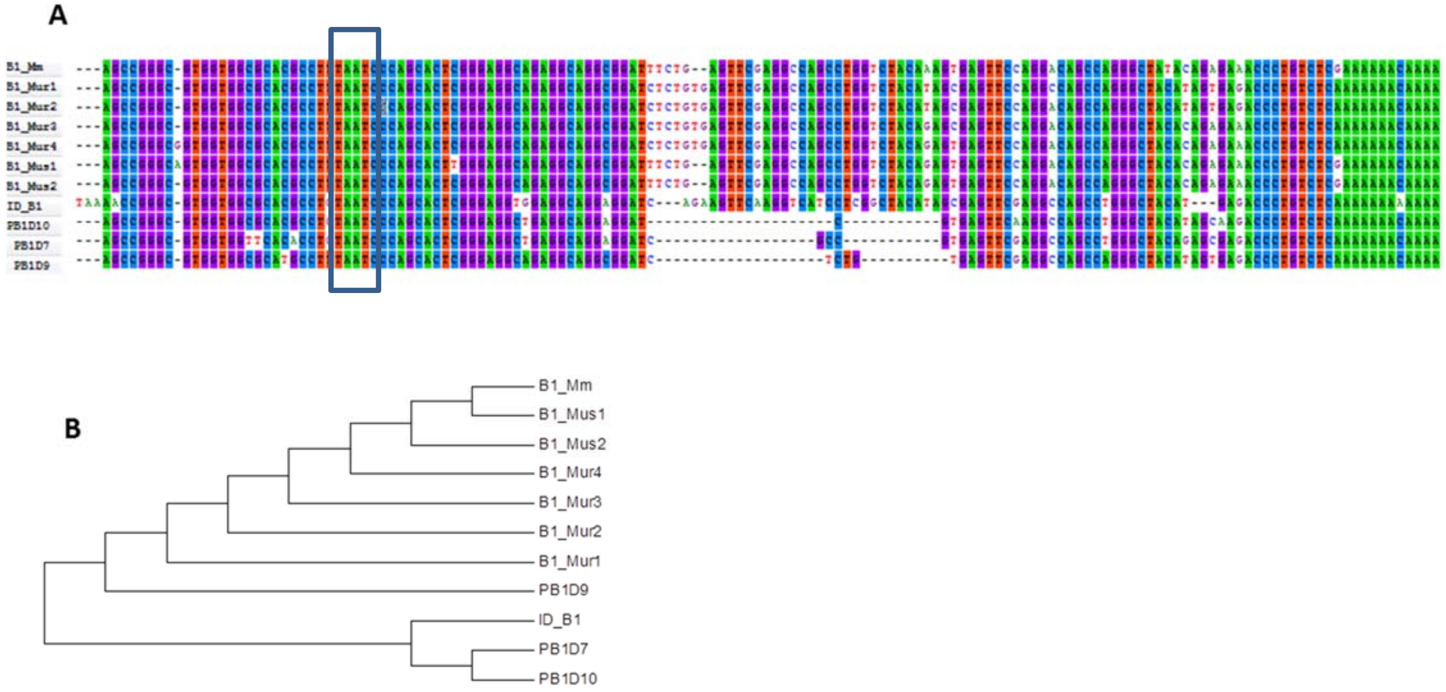

Figure 133. Alignment of Alu family repeats in mouse. A) Sequences are very similar. The "TAATC" motifs that could be bound by Obox transcription factors are highlighted. B) Phylogenetic tree.

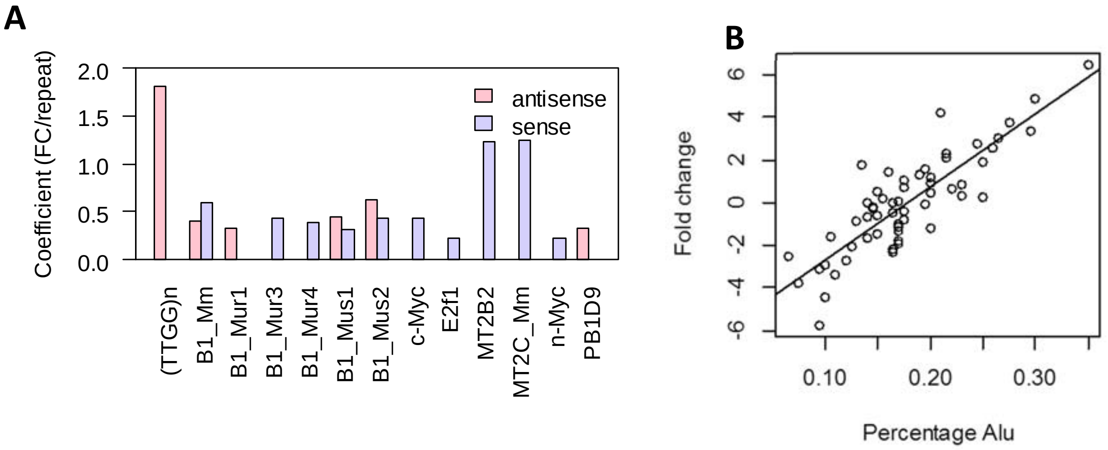

Figure 14. Confirmation using different mouse gene expression data of PD [5]. A) Significantly correlated repeats from regression analysis. B). Presents of Alu in gene groups predict fold-change during ZGA.

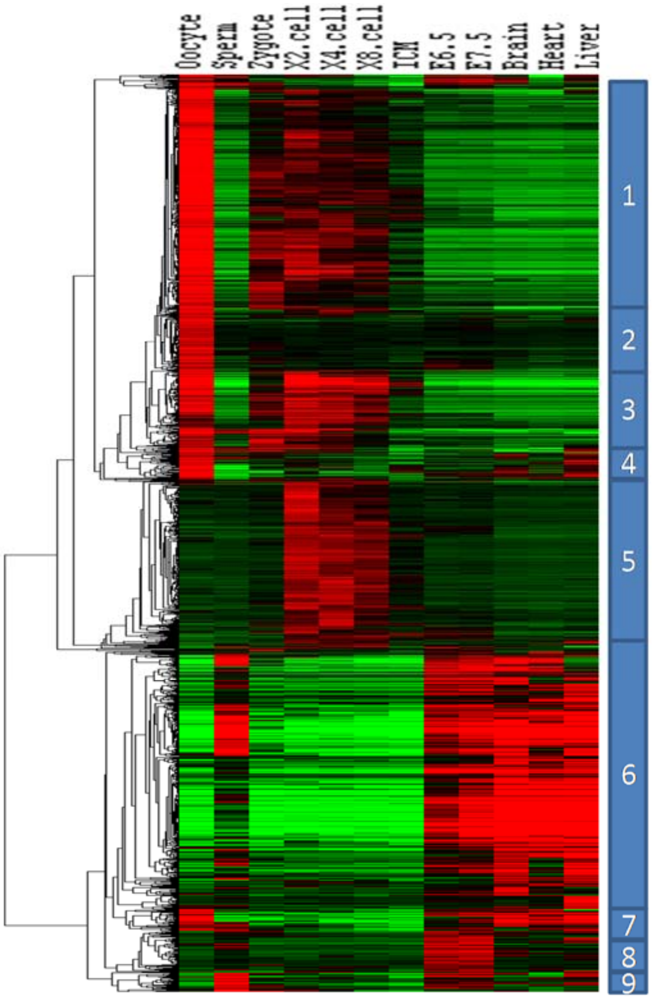

Figure 15. Change in methylation levels in promoter regions of 1495 genes. Data from Ref. [6]. Groups of genes show dynamic change in gene expression during early development. This pattern does not seem to correlate with the change in gene expression, as the gene groups show little overlap.

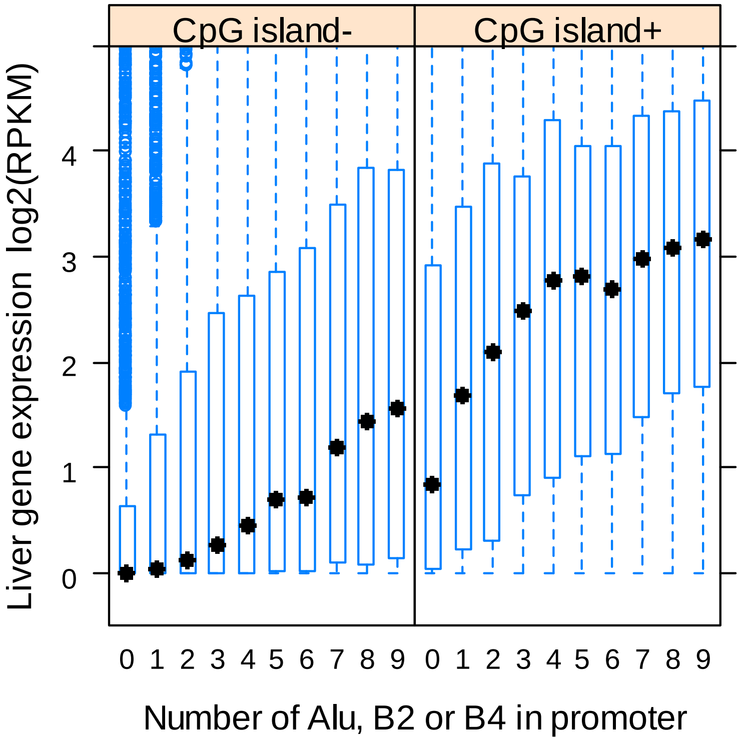

Figure 16. Expression levels in liver is higher in genes with CpG islands and multiple Alu family repeats in promoter region.

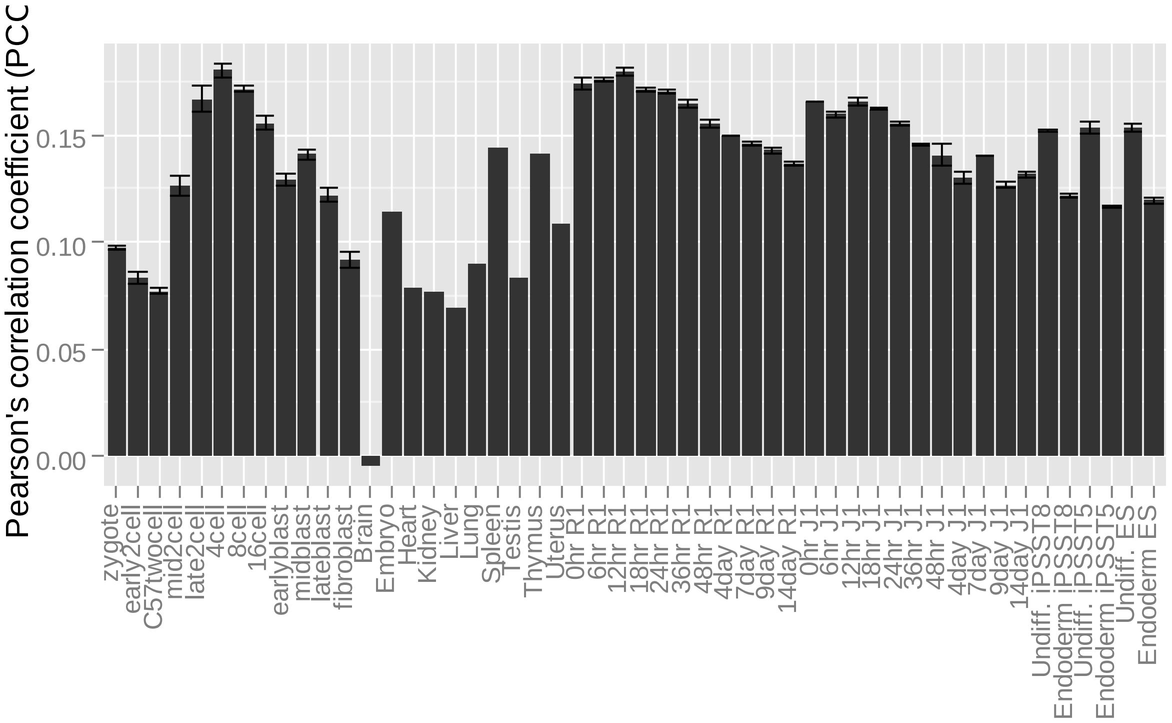

Figure 17. Correlation of the number of B2 family repeats in promoters and genome-wide gene expression.

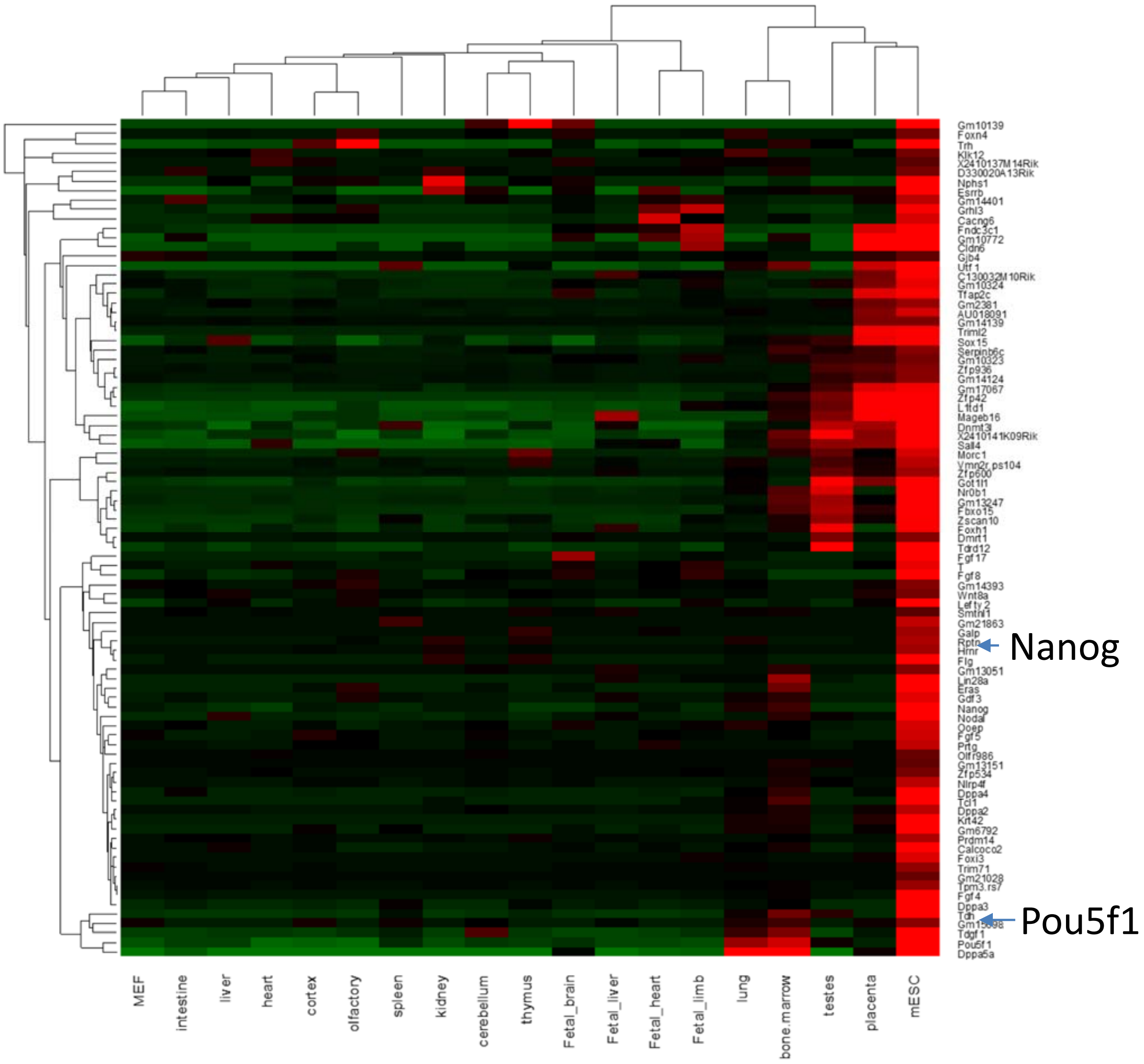

Figure 18. A group of mESC specific genes identified by k-means clustering. This is Cluster 13 in Fig. 6 in the main text.

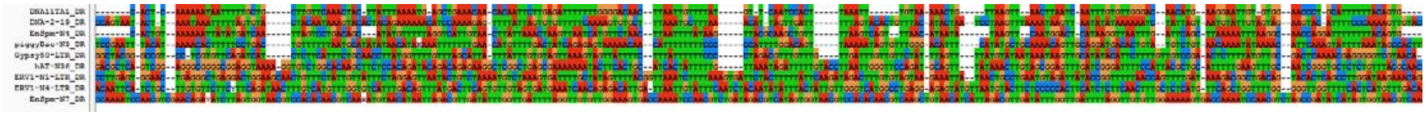

Figure 19. Repeats significantly associated with gene upregulation during ZGA in zebrafish are AT-rich sequences. Adenine (A) and thymine (T) are colored as red and green, respectively.

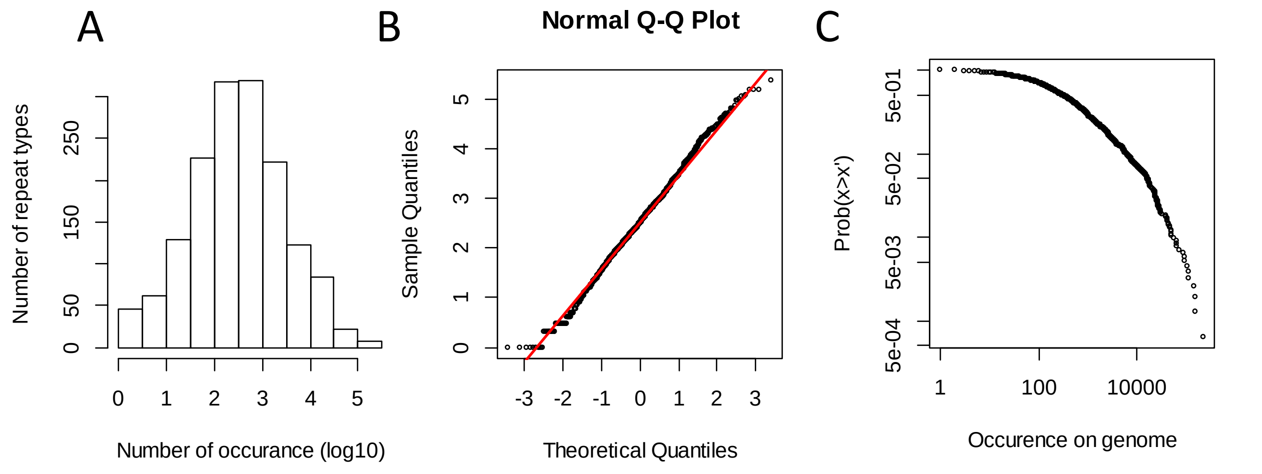

Figure 20. Distribution of repeats by number of occurrences on the genome. A) Distribution of the 1554 types of repeats according to how many times each repeat is observed in the mouse genome. After log-transformation, the distribution is bell-curved. B) The distribution is close to log-normal on a QQ plot. C) The distribution does not follow a power law.

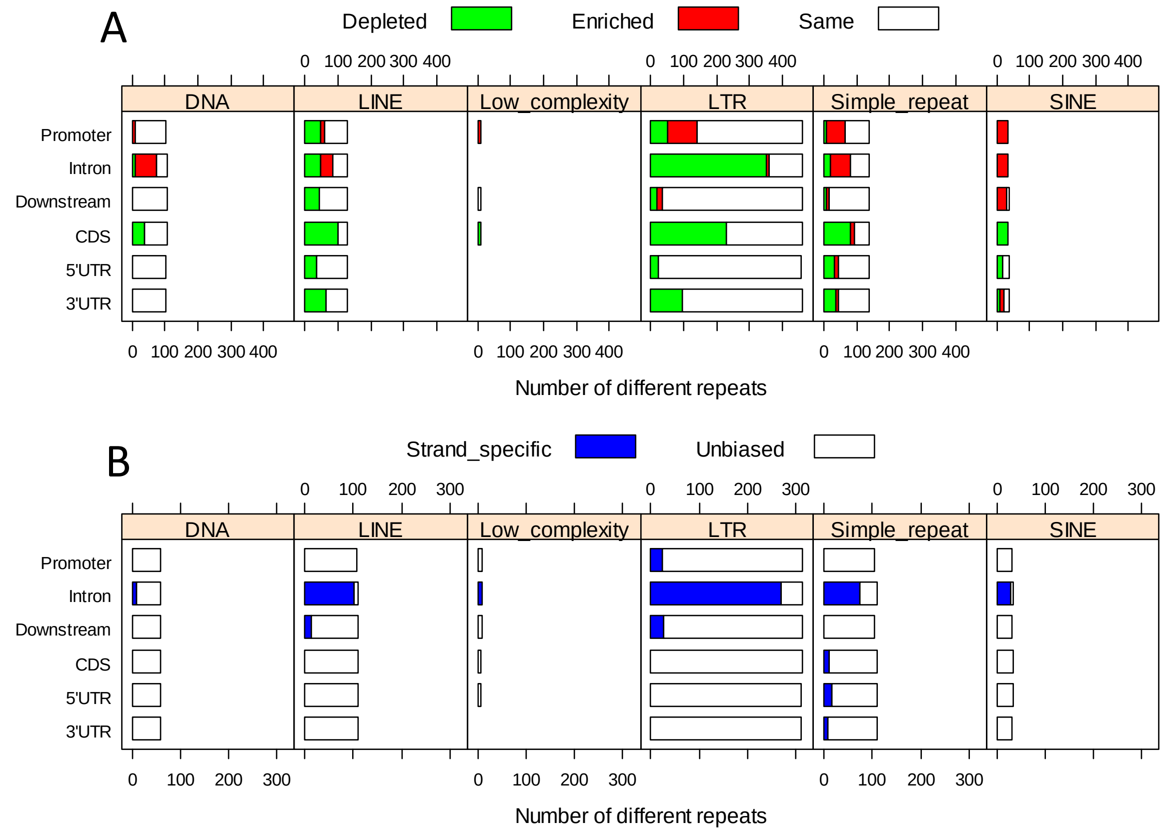

Figure 17. Distribution of repeats near genes. A) Enrichment of repeats in promoters and other regions. B) Strand-specificity of the repeats relative to genes. Note that the height of the bar represents the count of repeat types, not absolute repeat occurrence in the genome.

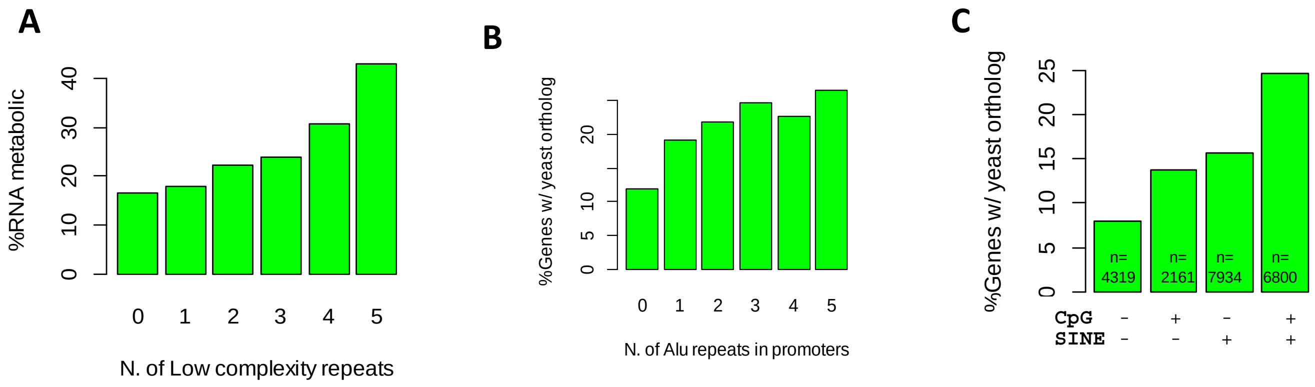

Figure 22. A) Low_complexity repeats are enriched in genes related to RNA metabolic process. B) Mouse genes with multiple Alu elements are more likely to have yeast orthologs in a dosage-dependent manner. C) The effect of SINE elements and the CpG islands are independent.

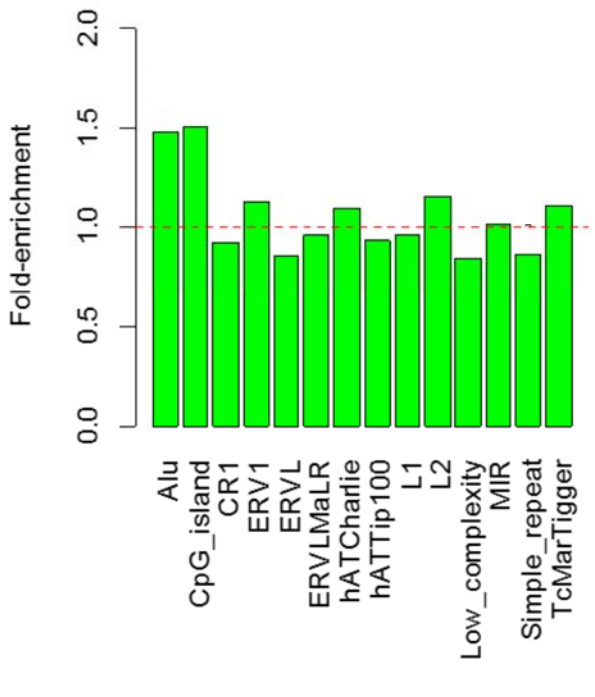

Figure 23. Human genes with Alu elements in promoters are more likely to have yeast orthologs.

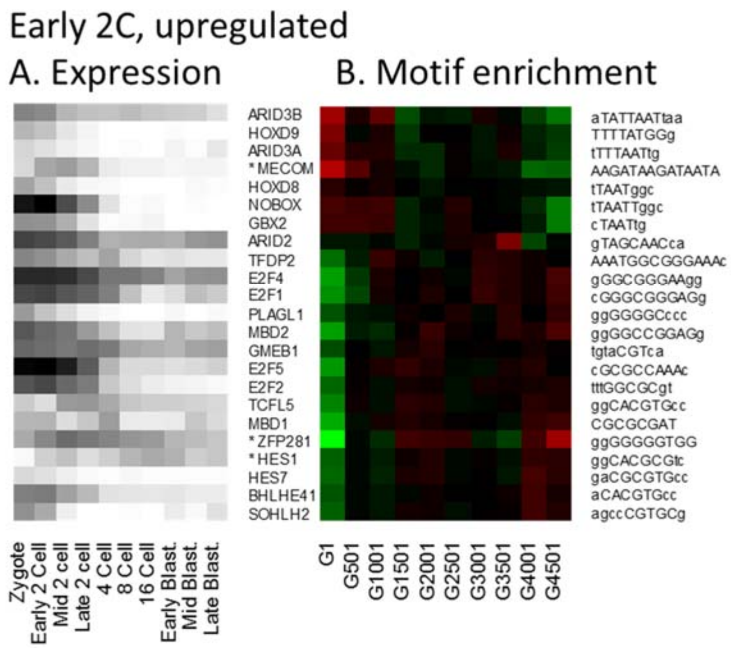

Figure 24. Enriched TF binding motifs in promoters of genes upregulated at early 2C stage. A. Expression levels of genes coding for TFs. Dark indicates higher expression. B. Corresponding TF binding motifs. Enrichment is shown in red and depletion in green.

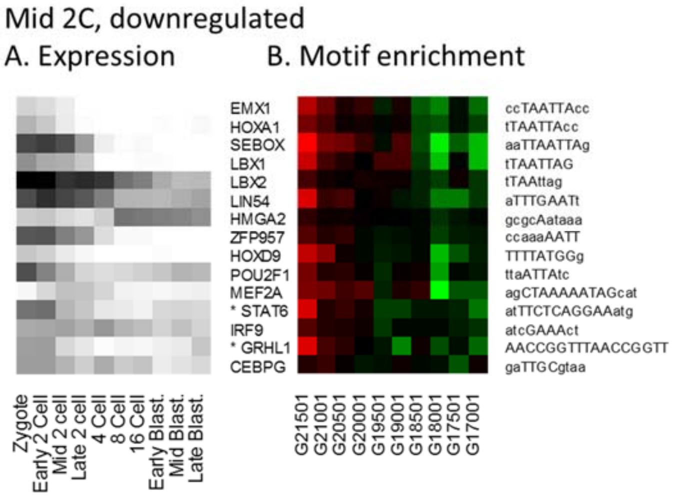

Figure 25. Enriched TF binding motifs in promoters of genes downregulated at mid 2C stage. A. Expression levels of genes coding for TFs. Dark indicates higher expression. B. Corresponding TF binding motifs. Enrichment is shown in red and depletion in green.

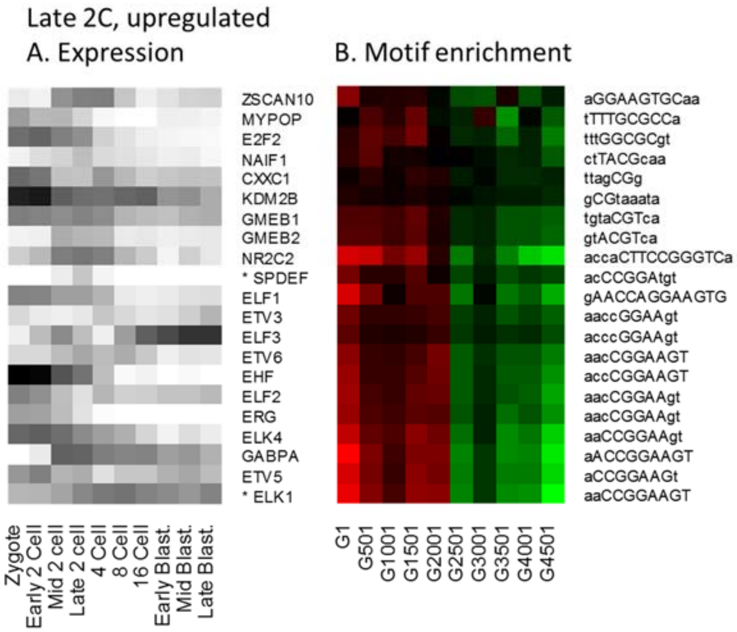

Figure 26. Enriched TF binding motifs in promoters of genes upregulated at late 2C stage. A. Expression levels of genes coding for TFs. Dark indicates higher expression. B. Corresponding TF binding motifs. Enrichment is shown in red and depletion in green.

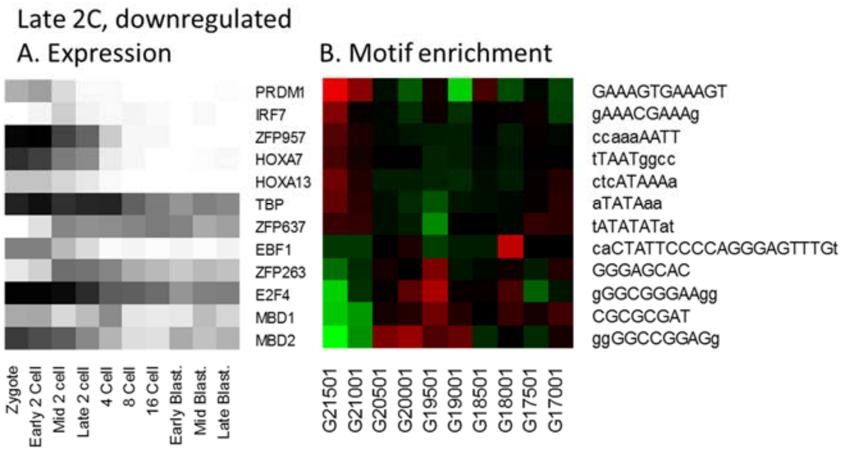

Figure 27. Enriched TF binding motifs in promoters of genes downregulated at late 2C stage. A. Expression levels of genes coding for TFs. Dark indicates higher expression. B. Corresponding TF binding motifs. Enrichment is shown in red and depletion in green.

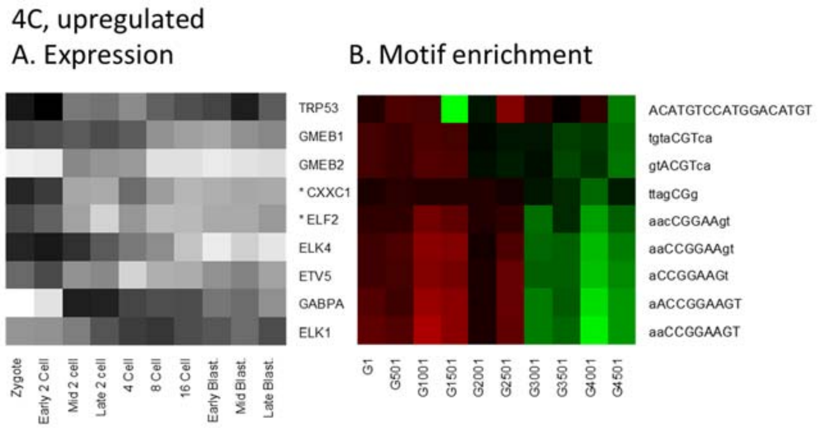

Figure 28. Enriched TF binding motifs in promoters of genes upregulated at 4C stage. A. Expression levels of genes coding for TFs. Dark indicates higher expression. B. Corresponding TF binding motifs. Enrichment is shown in red and depletion in green.

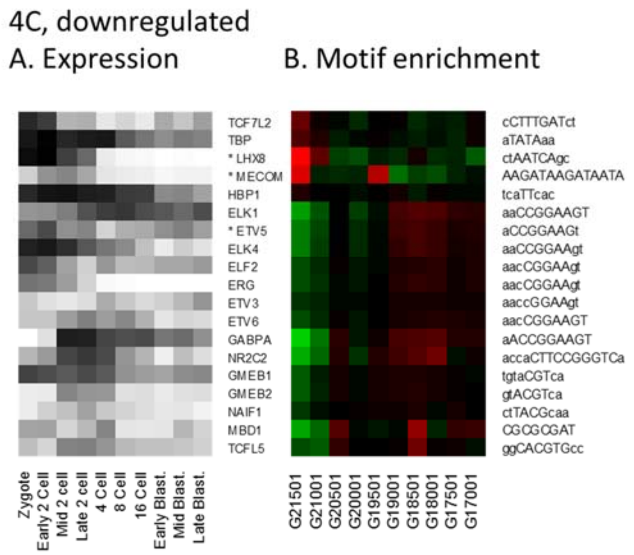

Figure 29. Enriched TF binding motifs in promoters of genes downregulated at 4C stage. A. Expression levels of genes coding for TFs. Dark indicates higher expression. B. Corresponding TF binding motifs. Enrichment is shown in red and depletion in green.

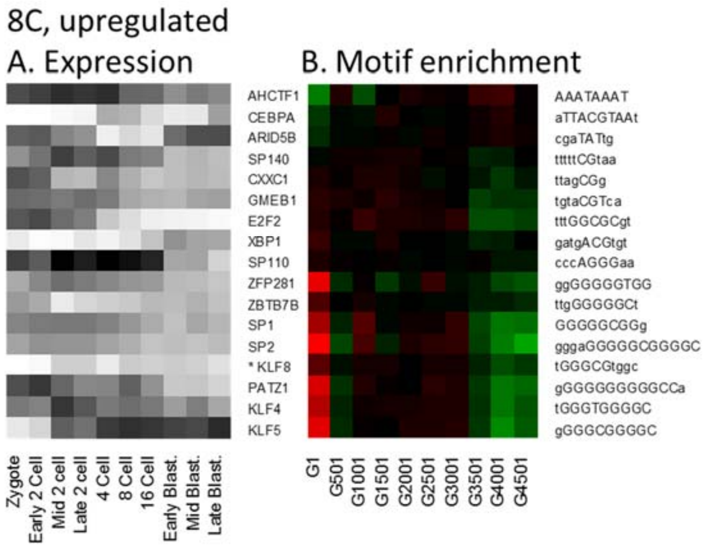

Figure 30. Enriched TF binding motifs in promoters of genes upregulated at 8C stage. A. Expression levels of genes coding for TFs. Dark indicates higher expression. B. Corresponding TF binding motifs. Enrichment is shown in red and depletion in green.

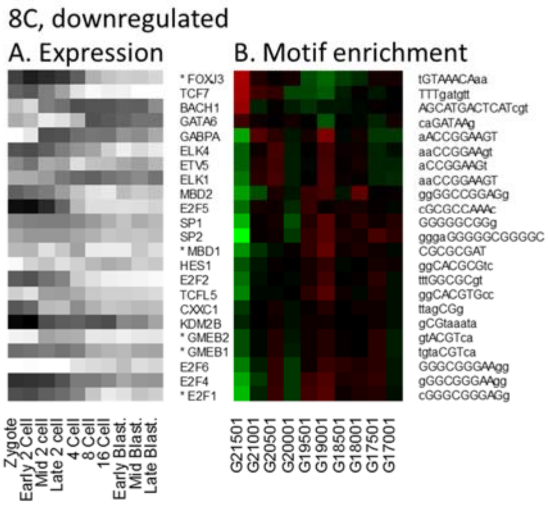

Figure 18. Enriched TF binding motifs in promoters of genes downregulated at 8C stage. A. Expression levels of genes coding for TFs. Dark indicates higher expression. B. Corresponding TF binding motifs. Enrichment is shown in red and depletion in green.

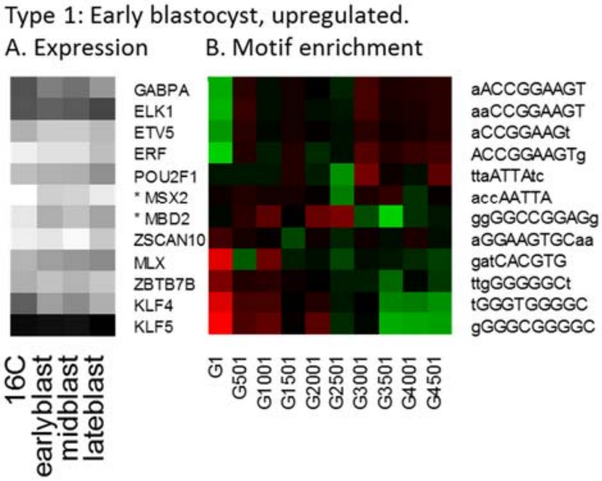

Figure 32. Enriched TF binding motifs in promoters of genes upregulated at early blastocyst stage in type 1 cells. A. Expression levels of genes coding for TFs. Dark indicates higher expression. B. Corresponding TF binding motifs. Enrichment is shown in red and depletion in green.

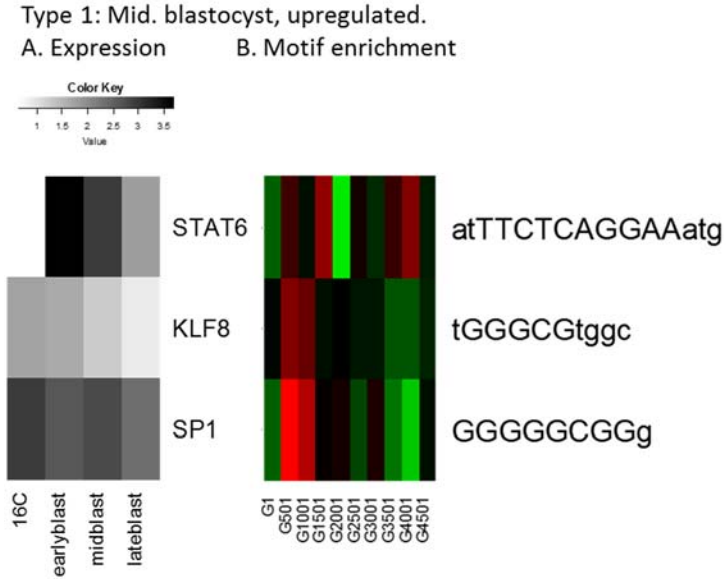

Figure 33. Enriched TF binding motifs in promoters of genes upregulated at mid blastocyst stage in type 1 cells. A. Expression levels of genes coding for TFs. Dark indicates higher expression. B. Corresponding TF binding motifs. Enrichment is shown in red and depletion in green.

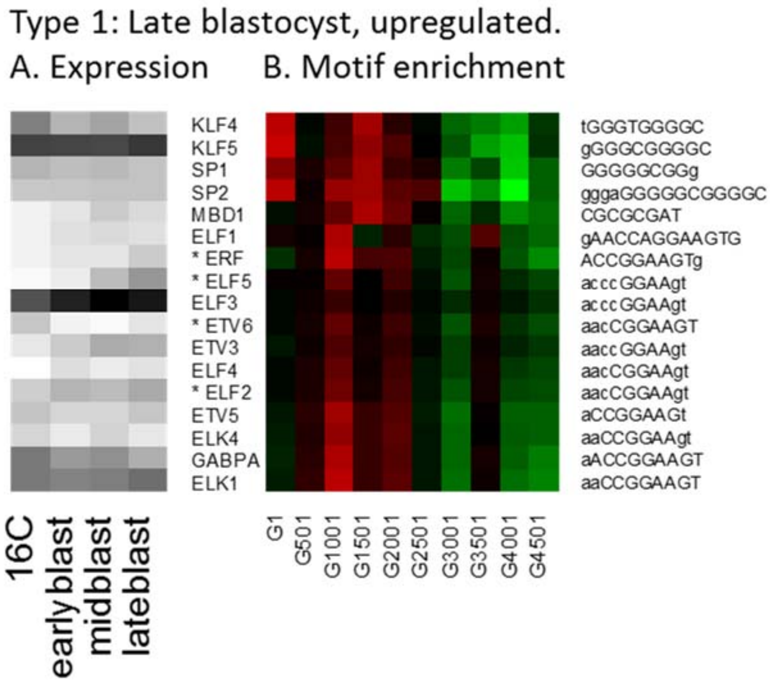

Figure 34. Enriched TF binding motifs in promoters of genes upregulated at late blastocyst stage in type 1 cells. A. Expression levels of genes coding for TFs. Dark indicates higher expression. B. Corresponding TF binding motifs. Enrichment is shown in red and depletion in green.

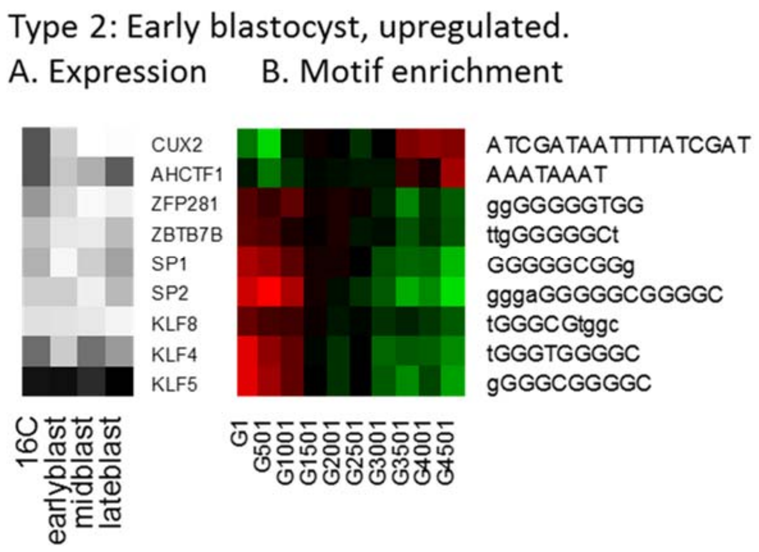

Figure 35. Enriched TF binding motifs in promoters of genes upregulated at early blastocyst stage in type 2 cells. A. Expression levels of genes coding for TFs. Dark indicates higher expression. B. Corresponding TF binding motifs. Enrichment is shown in red and depletion in green.

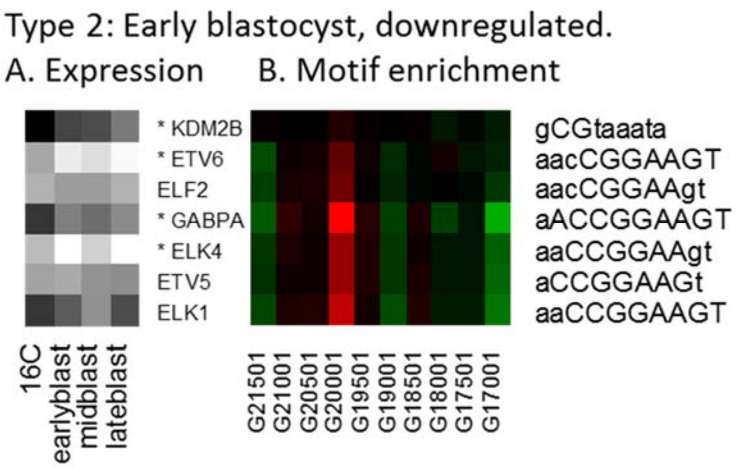

Figure 36. Enriched TF binding motifs in promoters of genes downregulated at early blastocyst stage in type 2 cells. A. Expression levels of genes coding for TFs. Dark indicates higher expression. B. Corresponding TF binding motifs. Enrichment is shown in red and depletion in green.

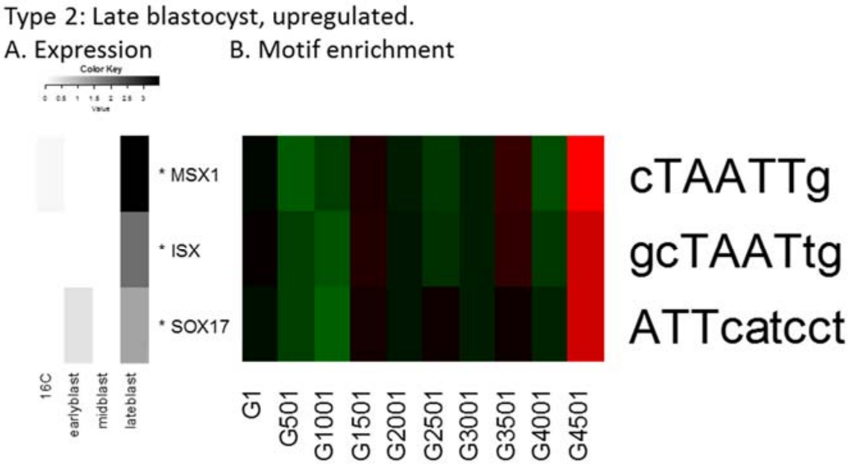

Figure 37. Enriched TF binding motifs in promoters of genes upregulated at late blastocyst stage in type 2 cells. A. Expression levels of genes coding for TFs. Dark indicates higher expression. B. Corresponding TF binding motifs. Enrichment is shown in red and depletion in green.

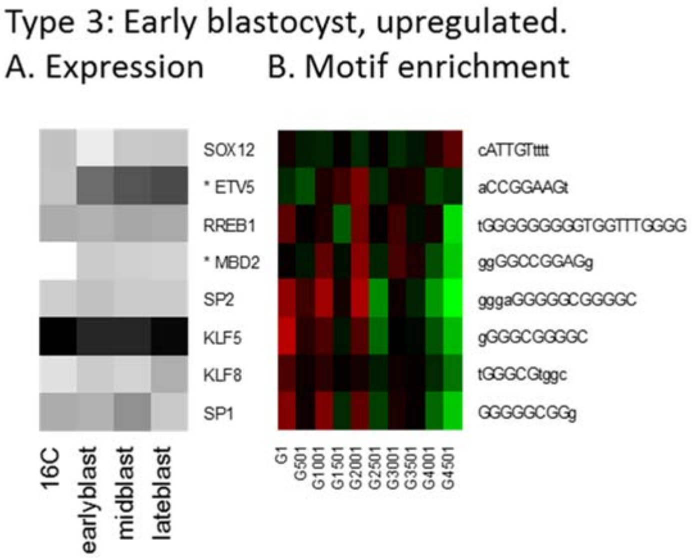

Figure 38. Enriched TF binding motifs in promoters of genes upregulated at early blastocyst stage in type 3 cells. A. Expression levels of genes coding for TFs. Dark indicates higher expression. B. Corresponding TF binding motifs. Enrichment is shown in red and depletion in green.

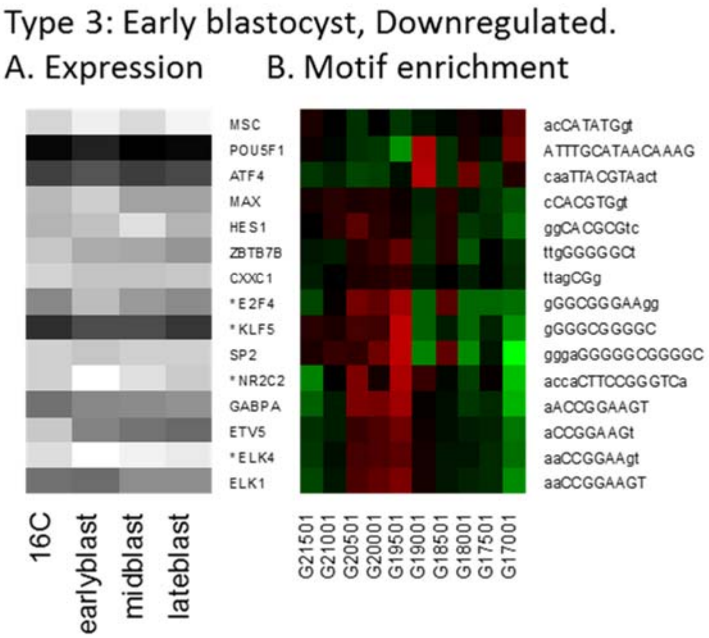

Figure 39. Enriched TF binding motifs in promoters of genes downregulated at early blastocyst stage in type 3 cells. A. Expression levels of genes coding for TFs. Dark indicates higher expression. B. Corresponding TF binding motifs. Enrichment is shown in red and depletion in green.

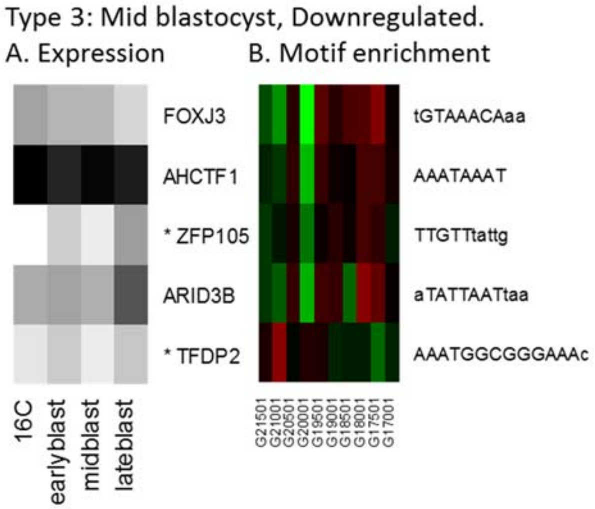

Figure 40. Enriched TF binding motifs in promoters of genes downregulated at early blastocyst stage in type 3 cells. A. Expression levels of genes coding for TFs. Dark indicates higher expression. B. Corresponding TF binding motifs. Enrichment is shown in red and depletion in green.

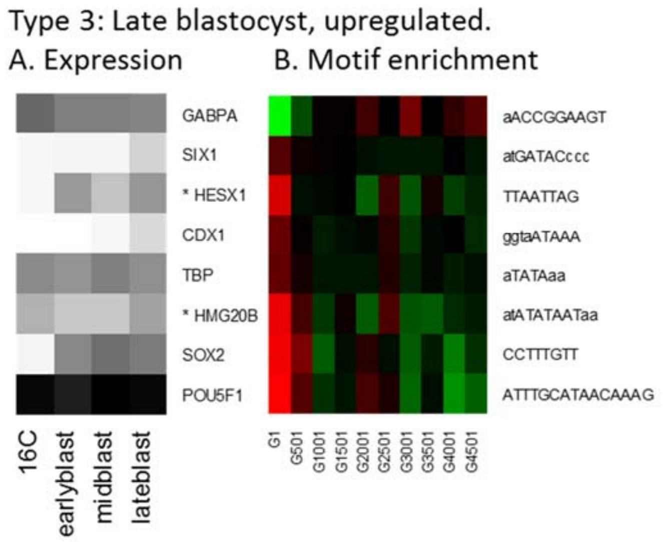

Figure41. Enriched TF binding motifs in promoters of genes upregulated at late blastocyst stage in type 3 cells. A. Expression levels of genes coding for TFs. Dark indicates higher expression. B. Corresponding TF binding motifs. Enrichment is shown in red and depletion in green.

1. Peaston, A.E., et al., *Retrotransposons regulate host genes in mouse oocytes and preimplantation embryos.* Dev Cell, 2004. **7**(4): p. 597-606.
2. Schoorlemmer, J., et al., *Regulation of Mouse Retroelement MuERV-L/MERVL Expression by REX1 and Epigenetic Control of Stem Cell Potency.* Front Oncol, 2014. **4**: p. 14.
3. Aanes, H., et al., *Zebrafish mRNA sequencing deciphers novelties in transcriptome dynamics during maternal to zygotic transition.* Genome Res, 2011. **21**(8): p. 1328-38.
4. Harvey, S.A., et al., *Identification of the zebrafish maternal and paternal transcriptomes.* Development, 2013. **140**(13): p. 2703-10.
5. Xue, Z., et al., *Genetic programs in human and mouse early embryos revealed by single-cell RNA sequencing.* Nature, 2013. **500**(7464): p. 593-7.
6. Smith, Z.D., et al., *A unique regulatory phase of DNA methylation in the early mammalian embryo.* Nature, 2012. **484**(7394): p. 339-44.
